# Identification of *Berberis* spp. as Alternate Hosts for *Puccinia achnatheri-sibirici* Under Controlled Conditions and Morphologic Observations of Sexual Stage Development of the Rust Fungus

**DOI:** 10.3389/fmicb.2020.01278

**Published:** 2020-06-25

**Authors:** Xinyao Ma, Yao Liu, Qiao Li, Xiaxia Tian, Zhimin Du, Zhensheng Kang, Jie Zhao

**Affiliations:** State Key Laboratory of Crop Stress Biology for Arid Areas, College of Plant Protection, Northwest A&F University, Yangling, China

**Keywords:** *Berberis* spp., gramineous grass, *Achnatherum*, alternate host, *Puccinia achnatheri-sibirici*

## Abstract

Gramineous grasses are a large group of species, many of which act as accessory (secondary) host for a large number of rust fungi, including devastating rust pathogens of cereals. Among the rust fungi, some are known to be heteroecious and have a complete macrocyclic life cycle with five types of spores on distinct plant species, but for many others the complete life cycle is unknown. *Puccinia achnatheri-sibirici*, a rust fungus infecting grasses in the genus *Achnatherum*, has been known for only its uredinial and telial stages; however, the other (pycnial and aecial) stages have not been identified. In this study, we demonstrate that *P. achnatheri-sibirici* is a heteroecious, macrocyclic fungus with the sexual stage on barberry (*Berberis* spp.) through inoculation. Pycnia and aecia were successively produced on the inoculated barberry plants. Inoculation of *Achnatherum extremiorientale* leaves with aeciospores produced yellow-orange uredinia with high infection types, whereas inoculation of wheat variety Mingxian 169, highly susceptible to *Puccinia striiformis* f. sp. *tritici* which causes stripe rust on wheat, produced chlorotic flecks but no uredinia. The ITS sequence analysis of *P. achnatheri-sibirici* did not match with any sequence in the NCBI database and had the highest homology with 94% compared to *Puccinia brachypodii* and *Puccinia aizazii*. Observations of the uredinial, telial, and basidial stages on *A. extremiorientale* and pycnial and aecial stages on *Berberis shensiana* using light and scanning electron microscopes revealed its characteristics. Morphological characteristics of urediniospores and teliospores are most similar with those of *P. achnatheri-sibirici* described in the literature. This study proved (1) the life cycle of *P. achnatheri-sibirici* as heteroecious and macrocyclic and the alternate host as barberry; (2) the description of life stages in the sexual cycle, especially the morphologies of aecial, pycnial, and basidial stages; and (3) the expansion of knowledge on the rust flora on barberry.

## Introduction

Gramineous plants around the globe harbor a large number of rust fungi with a wide range of morphologic and biologic differences (Anikster and Wahl, 1979). More than 380 species have been reported to belong to genera of *Puccinia* and *Uromyces* (Anikster and Wahl, 1979). Among the rust fungi on gramineous plants, some are important pathogens of cereal crops, such as *Puccinia striiformis* which causes stripe rust on wheat, barley, rye, triticale, and other cultivated or wild grasses; *Puccinia triticina* which causes leaf rust on wheat and grasses; *P. hordei* which causes leaf rust on barley; *Puccinia graminis* which causes stem rust on wheat, barley, rye, triticale, oats, and cultivated and non-cultivated grasses; and *Puccinia coronata* which causes crown rust on cereals and grasses.

Rust fungi belong to a group of obligate parasites that only depend on living plants. Many of them are heteroecious, requiring two unrelated plant species to complete their life cycle. Some heteroecious rust fungi with an asexual stage on gramineous grasses have a sexual stage on the alternate host barberry.

Barberry (*Berberis* spp.) plants are shrubs and can serve as alternate (aecial) hosts for several *Puccinia* species infecting cereals and gramineous grasses. So far, barberry spp. have been reported as an alternate host for *P. graminis* (de Bary, 1866; Roelfs, 1982), *P. striiformis* and *P. pseudostriiformis* (syn. *P. striiformis* f. sp. *poae*) (Jin et al., 2010), *Puccinia brachypodii* (Payak, 1965), *Puccinia minshanensis* (Zhuang and Wei, 1999), *P. brachypodii* var. *poae-nemoralis* (syn. *Puccinia arrhenatheri*, *Puccinia poae-nemoralis*) (Urban, 1967), *Puccinia pygmaea*, *Puccinia montanesis*, and *Puccinia brachypodii-phoenicoidis* (Cummins and Greene, 1966).

During an investigation on species and geographic distribution of barberry (*Berberis* spp.) plants and rusts on grasses in October 2013, abundant rust pustules in a mix of uredinia and telia were observed on leaves of a gramineous grass near Xinjie town, Baoji, Shaanxi, China (N34°41′30.09″, E106°52′16.23″; Elevation 1090 m). In November, 2015, uredinial and telial pustules on the same grass species were observed again in the same area. Because the grass plants were growing closely to barberry (*Berberis shensiana*) shrubs, and barberry plants have had heavy rust infection in the springs during our survey in this region since 2010 (Zhao et al., 2013; Wang et al., 2016), we postulated that the rust on the grass plants and rusts on the barberry plants might be related.

The grass plants were identified as *Achnatherum extremiorientale* (Hara) Keng ex P. C. Kuo and distributed in northeast, north, and northwest of China (Chu and Yang, 1990; Flora of China, e-version, website: http://www.iplant.cn/foc/). In the literature, the rust species *Puccinia achnatheri-sibirici* Wang was reported on several species of the genus *Achnatherum* as hosts, including *A. extremiorientale*, *Achnatherum sibiricum* (L.) Keng (Liu et al., 1991, 2015; Wang and Zhuang, 1998), and *Achnatherum splendens* (Trin.) Nevski (Wang et al., 2018). This rust has a narrow geographic distribution in the world, only in China and Japan (Wang and Wei, 1983, Wang et al., 2018). Teliospores of *P. achnatheri-sibirici* have a short pedicel or no pedicel, which is a distinguishable characteristics differentiating this species from any other rust fungi infecting *Achnatherum* and *Stipa* species (Wang and Zhuang, 1998). However, the rust pathogen was reported to have an incomplete life cycle with only uredinial and telial stages but other spore stages are unknown (Cummins, 1971; Wang and Zhuang, 1998).

The objectives of this study were to (1) determine if barberry is an alternate host for *P. achnatheri-sibirici* by artificial inoculation and (2) if so describe the morphological ontogeny of this rust fungus infecting *A. extremiorientale* and *B. shensiana* through light and scanning electron microscopy.

## Materials and Methods

### Plant and Pathogen

Plants and seeds of *A. extremiorientale* were collected at Xinjie town, Baoji, Shaanxi, China, in September 2014. Leaves of *A. extremiorientale* plants bearing uredinia and telia were sampled in the same region. Seeds of *A. extremiorientale* and wheat cv. Mingxian 169, which is highly susceptible to all races of *P. striiformis* f. sp. *tritici* (the wheat stripe rust pathogen) in China so far (Li and Zeng, 2002), were planted in plastic pots filled with soil mixture composed of turf, perlite, and vermiculite (Inner Mongolia Mengfei Biotech Co., Ltd., Hohhot, Inner Mongolia), and the plants were grown in a rust-free chamber of a greenhouse at 16–20°C and a diurnal 16-h light/8-h dark cycle.

Young plants of barberry (*B. shensiana*) collected from Taibai, Baoji, Shaanxi Province, were transplanted into pots filled with soil mixture and grown in the greenhouse at 16–20°C and a diurnal cycle of 16-h light/8-h dark.

To obtain isolates of the rust and increase urediniospores, a single uredinium of the grass leaf sample was transferred onto the surface of leaf of the grass *A. extremiorientale*. The inoculated grass were kept in a dew chamber at 10°C for 24 h in the dark and then moved to a growth chamber at 16–20°C with a diurnal cycle of 16-h light/8-h dark. Urediniospores of the single uredinium were used to increase spores by reinoculation.

### Inoculation of Wheat and *A. extremiorientale* Using Urediniospores of the Grass Rust

Seedlings of wheat cv. Mingxian 169 that had been grown for 10 days after planting and those of *A. extremiorientale* that had been grown for 30 days after planting were used for inoculation. Approximately 2-mg urediniospores were diluted by adding 2–3 drops (approx. 50–100 μL) of deionized water to make urediniospore suspension. Wheat and grass plants were inoculated using urediniospore suspension of *P. achnatheri-sibirici* and then kept for 24 h at 10°C in a dew chamber in the dark. The inoculated wheat plants were moved to a growth chamber with temperature set at 13 ± 3°C and a photoperiod of 16 h light/8 h dark. Infection types (ITs) on wheat plants were observed at 15–20 days post inoculation (dpi) using a 0–4 scale (Stubbs, 1985).

Urediniospores of the pure isolate from *A. extremiorientale* samples that produced low ITs (0, 0; or 1) on wheat but high ITs (3 or 4) on *A. extremiorientale* were multiplied on *A. extremiorientale* to produce an adequate amount of urediniospores in a greenhouse set at the same temperature and light conditions as described above for late use. A parallel test was conducted using *P. striiformis* f. sp. *tritici* race CYR32 as a positive control. IT “0” indicates no visible symptom, “0;” obvious necrosis or chlorosis without uredinia, and “1” obvious chlorotic or necrotic patches with a trance amount of uredinial sporulation. IT “2” describes large necrotic patches with a moderate amount of sporulation, IT “3” abundant sporulation with some necrosis or chlorosis, and IT “4” abundant sporulation with little or no chlorosis.

To produce teliospores, urediniospores from *A. extremiorientale*-derived isolates were used to inoculate *A. extremiorientale* plants at the adult plant stage at the conditions described above. Approximately sixty days after inoculation, leaves bearing telia were harvested and kept at 4°C for late use.

### Inoculation of *B. shensiana* With Basidiospores of the Grass Rust

Inoculation of barberry leaves with basidiospores was conducted following the procedure as previously described (Zhao et al., 2013). Leaves of *A. extremiorientale* bearing teliospores of *P. achnatheri-sibirici* within 10 days after harvest were wet–dry treated for 2–3 cycles at 16°C. In each cycle, the leaves bearing telia in a plastic box were soaked in water for 12 h and then dried under 16°C for 12 h. The leaves were cut into segments about 2 cm and placed on the surface of 2% (w/v) water agar medium in a Petri dish kept at 16°C. Teliospores were observed periodically for germination using a light microscope. Once teliospores began to germinate, the Petri dish was put over *B. shensiana* grown in a pot and kept for 3–4 days at 16°C in a dew chamber. After incubation, the inoculated *B. shensiana* plants were transferred to a growth chamber with the same conditions as mentioned above. Fertilization was performed by transferring nectar of one pycnium to another using a clean plastic inoculation loop.

The *B. shensiana* plant was covered for 1–2 days, and then the cover was removed. About 10–18 days after fertilization, aecial cups were excised with a sterile razor blade, and aeciospores were collected for inoculating *A. extremiorientale* and wheat plants.

### Inoculation of *A. extremiorientale* and Wheat Seedlings With Aeciospores of the Grass Rust

Seedlings of 30-day-old *A. extremiorientale* and 10-day-old wheat Mingxian 169 were inoculated using a water suspension of aeciospores produced on leaves of *B. shensiana* using the methods described above for inoculation using urediniospores. ITs were observed 15–20 days post inoculation.

### ITS Sequencing

Approximately 5 mg urediniospores produced on *A. extremiorientale* plants inoculated with aeciospores was placed into a 2-mL micorcentrifuge tube with two small steal balls ground to fine powder using a tissue grinding pestle (MGR 115, Sigma-Aldrich, MO, United States). Genomic DNA was extracted with Biospin Fungus Genomic DNA Extraction Kit (BioFlux, Tokyo, Japan). DNA concentration was measured by using a NanoDrop 2000 spectrophotometer (Thermo Fisher Scientific Inc., DE, United States). Genomic DNA was also extracted from an isolate of race CYR32 of *P. striiformis* f. sp. *tritici* in the same way. PCR amplifications of the grass rust DNA, together with DNA of the race CYR32 used as positive control and sterile ion-free water as a negative control, were performed with universal primer pairs ITS1RustF10d/ITS4 (White et al., 1990; Barnes and Szabo, 2007). PCR amplification was conducted according to the same method as described by Barnes and Szabo (2007). PCR products were electrophoresed in 1% (w/v) agarose gel in 1 × TAE buffer (0.04 M Tris–HCl, 0.001 M EDTA pH 8.0) at 3–5 v/cm for 40–60 min. The agarose gel was placed in a 0.1-μg/mL ethidium bromide (EB) solution for staining for 20 min and visualized using a Gel Imaging System (Bio-Rad ChemiDoc XRS, United States). The target band was excised from the gel, and DNA amplicons were collected using a Gel DNA Extraction Kit (BioTeke Corp., Beijing, China). The collected DNA amplicons were cloned by transduction into the pMD19 vector using a T-vector pMD19 (simple) Kit [Takara Biotechnology (Dalian) Co., Ltd, Japan] and sequenced by Aoke Biotechnology Co., Ltd. (Beijing, China). We sequenced a single clone for each type spore and repeated it three times. For each type of spore, we choose one of the repeats for sequence analysis. All the sequences of *P. achnatheri-sibirici* were deposited in GenBank under accession numbers MN913585, MN913586, and MN913598, while the accession numbers deposited in GenBank of *P. brachypodii* (on *Brachypodium distachyon*) analyzed in this study were MN915123 and MN915138. The datasets analyzed for this study can be found in the NCBI GenBank^1^.

DNA sequences were blasted at the NCBI database (see footnote 1) for determination of the rust fungus. Sequence alignment was implemented using the BioEdit program^2^. Phylogenetic tree procedures of *P. achnatheri-sibirici* and other species in the genus *Puccinia* downloaded from the NCBI database (Table 1) were constructed based on ITS sequences using maximum parsimony analysis in software PAUP^∗^4.0b10 (Swofford, 2002).

**TABLE 1:** Sequence analysis for identification of *Puccinia achnatheri-sibirici.*

***Puccinia species***	***Host***	***GenBank accession***
*P. achnatheri-sibirici*	*Achnatherum extremiorientale*	*MN913585 (teliospores, this study)*
*P. achnatheri-sibirici*	*A. extremiorientale*	*MN913586 (urediniospores, this study)*
*P. achnatheri-sibirici*	*Berberis* spp.^a^	*MN913587 (aeciospores, this study)*
*P. aizazii*	*Jasminum humile*	*KY386658*
*P. brachypodii*	*-*^b^	*GQ457303*
*P. brachypodii*	*Brachypodium distachyon*	*MN915132 (H-KI, this study)*
*P. brachypodii*	*B. distachyon*	*MN915138 (F-CO, this study)*
*P. bromina*	*Bromus sp.*	*DQ460719*
*P. coronata*	*Agropyron sp.*	*HM131253*
*P. gansensis*	*A. inebrians*	*HM057115*
*P. graminis*	*Agropyron repens*	*AF468044*
*P. holcina*	*Holcus lanatus*	*DQ513000*
*P. hordei*	*Vulpia myuros*	*HQ317527*
*P. pseudostriiformis*	*Poa pratensis*	*HM057134*
*P. striiformis*	*T. aestivum*	*HM057118*
*P. striiformis*	*T. aestivum*	*HM057119*
*P. striiformis*	*Hordeum secalinum*	*HM057124*
*P. striiformis*	*Elymus elymoides*	*HM057130*
*P. striiformis*	*T. aestivum*	*HM057132*
*P. striiformoides*	*Dactylis glomerata*	*HM057109*
*P. striiformoides*	*D. glomerata*	*HM057137*
*P. triticina*	*T. aestivum*	*EU014050*
*Uromyces dactylidis* (outgroup)	*D. glomerata*	*HM057148*

*^*a*^*Berberis* spp., *B. shensiana*, *B. cavalerie*, and *B. aggregate* were identified as alternate hosts for *P. achnatheri-sibirici* in this study. *B. shensiana* was used for molecular analysis. ^*b*^“-” No information available at NCBI database.*

### Morphology of *P. achnatheri-sibirici*

To observe the morphological characteristics of *P. achnatheri-sibirici* spores, leaf samples of uredinia and telia were harvested from *A. extremiorientale*, together with leaf samples of pycnia and aecia from barberry. Approximately 3-cm *A. extremiorientale* leaf segments with uredinia and/or telia and 2 cm × 3 cm barberry leaf pieces with pycnia or aecia were detached. All leaf samples from both hosts were used for histological observations using a light and/or electron microscope. Leaf sections were fixed in phosphate buffer (pH 7.4) of a final concentration of 0.1 M with addition of 2.5% (v/v) glutaraldehyde at 4°C overnight. The samples were then dehydrated using a series of grade ethanol solution and substituted with isoamyl acetate. After CO_2_ critical-point drying, the samples were sputter coated with wolfram and observed using a scanning electron microscope (SEM; Hitachi S-4800, Tokyo, Japan) at 10 kV.

Urediniospores and teliospores of *P. achnatheri-sibirici* were scratched from leaf segments and put into a drop (10–20 μL) of ion-free water on a glass slide with a glass cover slip and observed morphologically using a light microscope (Olympus BX53, Tokyo, Japan). We measured the length, width, wall thickness, apex thickness, and pedicels of teliospores and the size of basidiospores by software cellSens Imaging coupled with a microscope. Urediniospores and teliospores were observed morphologically and identified according to recorded gramineous rust species (Wang and Wei, 1983).

Treatments of teliospore germination to produce basidiospores were conducted according to the method described by Zhao et al. (2013).

## Results

### Morphology of *A. extremiorientale* and Rust Infection

**FIGURE 1:**
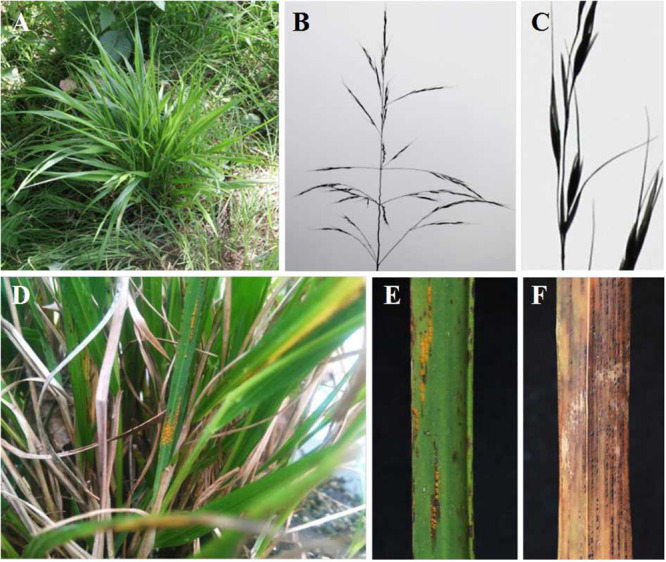
Morphology of gramineous grass, *Achnatherum extremiorientale*, and natural rust infection on the grass species by *Puccinia achnatheri-sibirici*, observed in Xinjie, Baoji, Shaanxi Province, in October 2013. **(A–C)** Plants, heads, and seeds of *Achnatherum extremiorientale*. **(D–F)** Uredinial and telial symptoms of natural infection of *P. achnatheri-sibirici* on grass *A. extremiorientale*, respectively.

*Achnatherum extremiorientale* is a perennial gramineous grass, belonging to the tribe of Stipeae, the subfamily of Pooideae, and the family of Gramineae. This grass grows in a cluster (Figure 1A) and has a wide long leaf (up to 50 cm) often rolling upward at the margin, panicle with 3–6 branches and 20–40 cm in length, and fusiform caryopsis approximately 4 mm in length (Figures 1B,C).

Under natural conditions, *A. extremiorientale* plants infected with *P. achnatheri-sibirici* produced yellow- to brown-colored uredinia on both sides of a leaf we observed in fall (Figure 1D). The uredinia broke through the epidermis of the infected leaves presenting powdery pustules with obvious black necrosis around/on uredinia (Figure 1E). During the development of uredinia, black telia were produced and embedded underneath the epidermis (Figure 1F).

### *B. shensiana* Serves as an Alternate Host for *P. achnatheri-sibirici*

**FIGURE 2:**
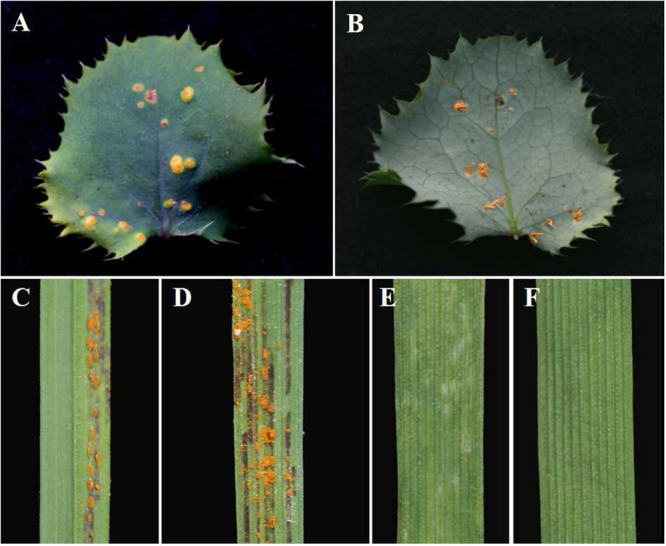
Pycnial and aecial stages of *Puccinia achnatheri-sibirici* on barberry (*Berberis shensiana*) after infection by basidiospores and responses of *Achnatherum extremiorientale* and wheat cv. Mingxian 169 to aeciospores. **(A)** Pycnial stage of *P. achnatheri-sibirici* infecting the barberry (*B. shensiana* Ahrendt) after inoculation with basidiospores. **(B)** Aecial formation on the infected barberry. **(C)** The grass, *Achnatherum extremiorientale*, presenting orange uredinia on leaves after inoculation using *P. achnatheri-sibirici* aeciospores produced on the barberry in Figure 2B. **(D)** Black stripes formed and restricted between leaf veins of the grass. **(E)** Wheat cv. Mingxian 169 showed obvious chlorotic lesions after inoculation with aeciospores of *P. achnatheri-sibirici*. **(F)** Non-inoculated leaves of Mingxian 169 wheat used as control.

After inoculation of *B. shensiana* with basidiospores that are produced from germinated teliospores of *P. achnatheri-sibirici*, pycnia appeared on the surface of the leaves at 7–9 dpi (Figure 2A). After fertilization, orange-colored aecial cups, variable in number, began to appear on the opposite side of the leaf from the pycnia at 11–16 dpi (Figure 2B).

Inoculation of *A. extremiorientale* and wheat Mingxian 169 plants with aeciospores produced orange-colored uredinia accompanied with longitudinal black stripes around the pustules (Figures 2C,D). Symptoms produced by artificial inoculation with aeciospores were similar to that of rust infection on the grass under natural conditions (Figure 1E). However, inoculation of Mingxian 169 with aeciospores produced only chlorotic necrosis lesions with IT 0; (Figure 2E) in contrast to healthy leaves of wheat (Figure 2F).

### Molecular Data

**FIGURE 3:**
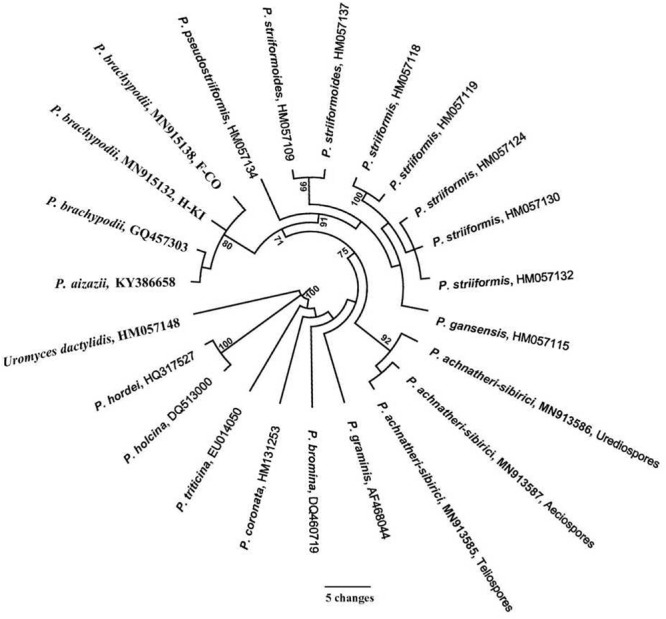
Phylogenetic tree of *Puccinia achnatheri-sibirici* and *Puccinia* species based on the sequence of nuclear ribosomal internal transcribed spacer (ITS) amplified with primer ITS1RustF10d/ITS4 (White et al., 1990; Barnes and Szabo, 2007). Clade robustness was assessed using a bootstrap analysis with 1,000 replicates. The numbers on the branches are bootstrap values from MP. Reference sequences used in this analysis are listed in Table 1.

The ITS region of *P. achnatheri-sibirici* amplified with ITS1RustF10d/ITS4 primers produced a 541-bp amplicon. BLAST research of the NCBI database^3^ did not identify any sequences sharing above 95% homology. Top five *Puccinia* species sharing relatively high homologies included *Puccinia aizazii* (on *Jasminum humile*, 94%), *P. brachypodii* (92% or 94%), *P. striiformis* (on *Dactylis glomerata*, 92%), *P. striiformoides* (92%), and *P. striiformis* f. sp. *tritici* (on wheat, 91%). Phylogentic analysis of the ITS region indicated that teliospores on leaves of *A. extremiorientale*, aeciospores produced on *B. shensiana*, and urediniospores produced on the grass inoculated with the aeciospores were clustered into a clade with a bootstrap value of 92%, strongly distinguished from the other rust species used in the present study (Figure 3).

Based on the tree in Figure 3, *P. brachypodii* is more closely related to the *P. striiformis* species complex. So, a cross-validation experiment was conducted. Two *P. brachypodii* isolates H-KI and F-CO which infect *B. distachyon* were used to inoculate grass *A. extermiorientale*, producing low infections with merely light necrosis. Similarly, *B. distachyon* represented a highly resistant reaction to *P. achnatheri-sibirici* after inoculation. This result indicated that the rust pathogen *P. achnatheri-sibirici* is not *P. brachypodii*, supporting the results of *P. achnatheri-sibiri* and *P. brachypodii* grouped into two subclades (Table 1 and Figure 3).

### Spore Development and Morphology

**FIGURE 4:**
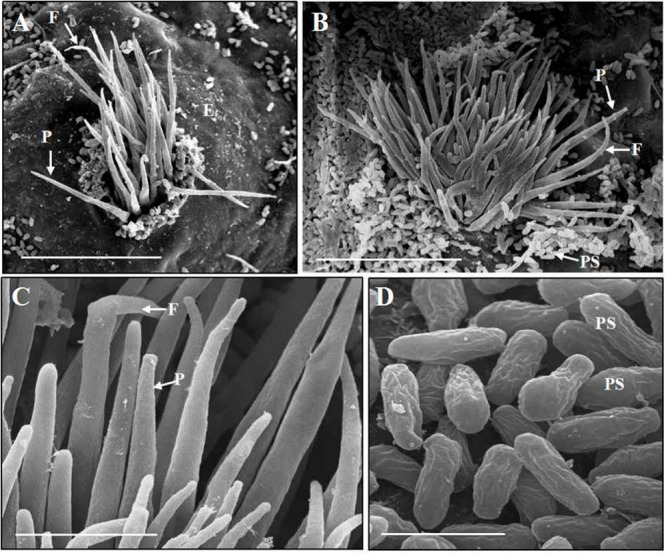
Scanning electron microscopy observations of *Puccinia achnatheri-sibirici* infecting *Berberis shensiana* Ahrendt at the pycnial stage. **(A)** A young pycnium at the early stage with eruption from epidermis (E) of the host, producing paraphyses (P) and flexuous hyphae (F). Bar = 50 μm. **(B)** Paraphyses (P) and flexuous hyphae (F) of a mature pycnium radiated on the ostiole of the pycnium with production of numerous pycniospores congesting around the opening. Bar = 50 μm. **(C)** Enlargement of the paraphyses (P) and flexuous hyphae (F). Bar = 5 μm. **(D)** Mature pycniospores (PS). Bar = 5 μm.

After inoculation, *B. shensiana* leaves infected with *P. achnatheri-sibirici* basidiospores produced light yellow lesions 7–9 dpi, which continue to expand to about 3–5 mm in diameter after 18–20 dpi (Figure 2A). Simultaneously, pycniospores, paraphyses, and flexuous hyphae were produced in pycnia under the epidermis of barberry leaves, subsequently broke through the epidermis (Figure 4A), and congested around the ostioles of pycnia as the production of numerous pycniospores in succession (Figure 4B). The paraphyses and flexuous hyphae radiated to separate during the development of pycnia (Figures 4B,C). Mature pycniospores were oval and smooth-surfaced, 4.3 (3.5–5.0) μm-1.5 (1.3–1.7) μm in size (Figure 4D). Macroscopically, drops of hyaline nectars (honeydews) were formed on the ostioles.

**FIGURE 5:**
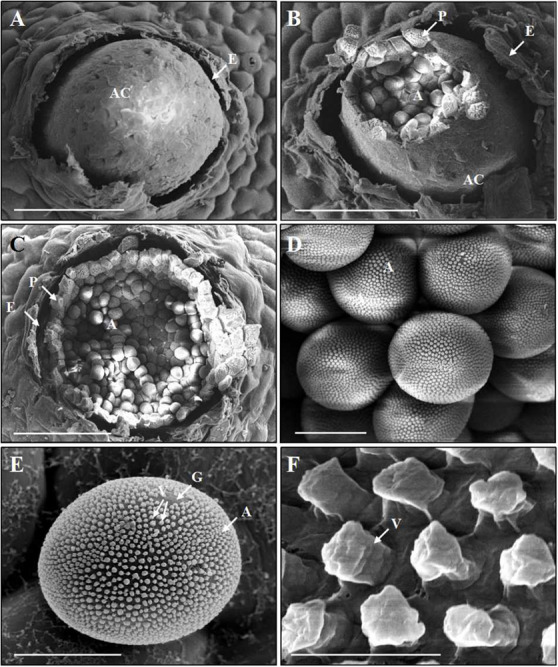
Scanning electron microscopy observations of the development of aecia and aeciospores of *Puccinia achnatheri-sibirici* infecting *Berberis shensiana* at the aecial stage. **(A)** An initial aecial cup (aecium, A) erupted from the epidermis (E) of the host. Bar = 100 μm. **(B,C)** A mature aecial cup (AC) broke and released aeciospores (A) embraced by a layer peridium (P) that was formed by peridial cells. Bar = 100 μm. **(D,E)** A mature aeciospore (A) was spherosome-shaped and has germ pores (G) and the numerous, column-shaped ornaments (verrucae, V) on the surface. Bar = 10 μm. **(F)** Close-up of verrucae (V). Bar = 1 μm.

After fertilization, young aecia formed on the opposite surface, about 20 dpi (Figure 5A). Young aecia first appeared as dome-shaped and then developed into long finger-shaped horns (Figures 2B, 5B,C). As the aecium matured in the peridium, it split open and released aeciospores (Figure 5B). A ripe aeciospore was spherosome-shaped, having several germ pores and the ornaments (verrucae) consisting of a great number of short columns on the surface (Figures 5D–F).

**FIGURE 6:**
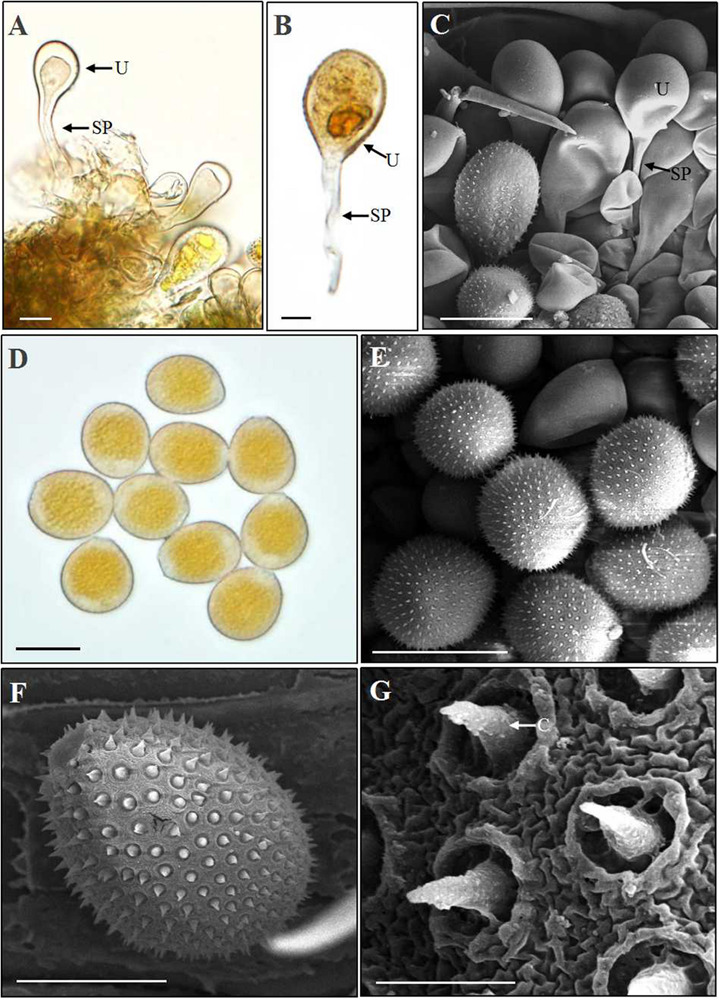
Light and scanning electron microscopy observations of urediniospores of *Puccinia achnatheri-sibirici.* **(A–C)**. A young urediniospore (U) developing at the top of a long sporophore (SP) formed in a uredinium based on observations using light and scanning electron microscopy. Bar = 20 μm. **(D)** Light microscopy observation showing orange and near sphere-shaped urediniospores. Bar = 20 μm. **(E)** Scanning electron microscopy (SEM) observation presenting urediniospore production in a uredinium. Bar = 20 μm. **(F)** SEM observation showing a mature urediniospore with numerous cones on the surface. Bar = 10 μm. **(G)** Enlargement of the cones (C) on the surface of a urediniospore. Bar = 1 μm.

After 18–25 dpi, aeciospores infected *A. extremiorientale* leaves to produce yellow uredinia (Figures 2C,D). Light and scanning electron microscopic observations showed that young urediniospores differentiated from the apex of long sporophores and surface-smoothed at the early stage (Figures 6A–C), becoming orange-colored, near sphere-shaped, mature urediniospores with numerous ornaments on the surface (Figures 6D–F). The ornament was broken through the surface of a urediniospore to produce a cone (thorn) in shape beyond the surface. The cone towered in the middle of the opening, looking like a crater of a volcano in shape (Figure 6G).

**FIGURE 7:**
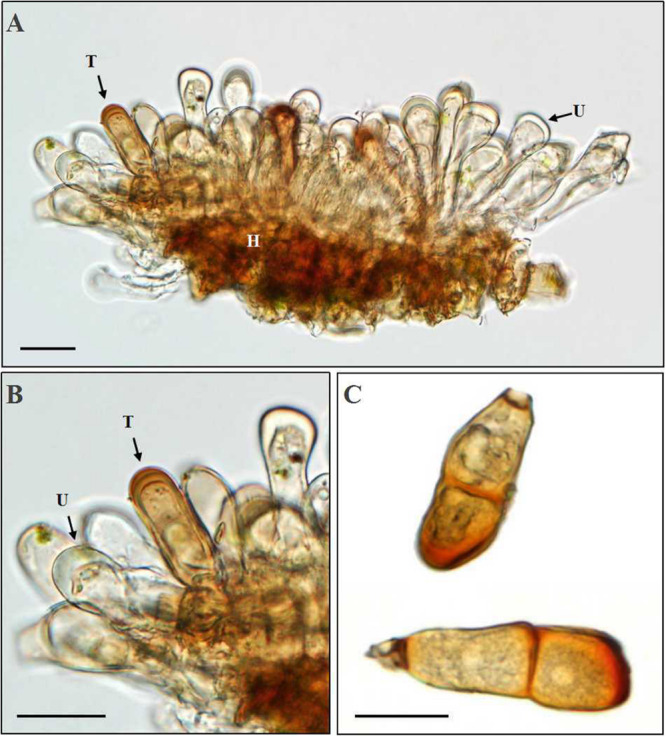
Morphologic observation of *Puccinia achnatheri-sibirici* teliospores by light microscopy. **(A,B)** Production of a teliospore (T) in a uredinium (U). Bar = 20 μm. **(C)** Mature teliospores with/without a short stalk cell. Bar = 20 μm.

At the late stage of uredinial development, hyphae growing in a uredinium began to differentiate to form young dark-brown teliospores (Figures 7A,B). The young teliospores continuously developed to give rise to mature teliospores, which were two-celled and had or had no constriction at the connection (septum) of the two cells and are dark brown in color. The color of the upper cell was darker than that of the lower cell.

Based on the data of measurement of 105 individual teliospores, teliospores generally were two-celled, with a short pedicle and brown-colored. The color of the upper cell of a teliospore was darker than that of the lower cell. The top of teliospores were nearly domed with a thick and dark-brown cell wall. The two-celled teliospores were 40.84 (27.64–57) μm long and 20.46 (7.83–29.11) μm wide. The thickness of the side cell wall were 1.73 (0.52–3.43) μm, and that of the apical cell wall was 4.07 (1.63–6.28) μm. Pedicels were short and averaged 4.85 (0.84–8.36) μm in length and light brownish in color. Sometimes, one-celled teliospores could be observed under microscopic observations (Figure 7C). Morphologic comparisons were made between with teliospores of the rust and species of the genus *Puccinia*-infecting gramineous plants described by Cummins (1971). The rust pathogen infecting the grass *A. extremiorientale* in morphological characteristics, similar to *P. achnatheri-sibirici* descried by Wang and Wei (1983) and Cummins (1971), was confirmed as *P. achnatheri-sibirici*. The differences among the rust fungi on teliospore morphology infecting *Achnatherum* and *Stipa* are shown in Table 2.

**TABLE 2:** The characteristics of teliospores of different rust fungi infecting *Achnatherum* and *Stipa*.

***Puccinia* species**	**Host**	**Size (μm)**	**Apex thickness (μm)**	**Pedicel (μm)**
*P. achnatheri-sibirici^a^*	*A. extremiorientale*	27.64–57 ×7.83–29.11	1.63–6.28	0.48–8.36
*P. achnatheri-inebriantis*	*A. inbrians*	36–57 × 15–21	9–12	45–70
*P. achnatheri-sibirici*	*A. extremiorientale*	35–58 ×15–20	2.5–5	-^b^
	*A. sibiricum*			
*P. burnettii*	*Stipa* sp.	31–40 ×20–24	3.5–5	200
*P. graminis*	*A. splendens*	38–60 × 18–25	5–13	-^c^
*P. stipae*	*A. splendens*	38–63 × 18–23	8–12	100
	*S. breviflora*			
*P. stipa-sibiricae*	*A. extremiorientale*	30–48 × 16–22	6–12	200
	*A. inebriana*			
	*A. sibiricum*			
	*Stipa* sp.			

*^*a*^*P. achnatheri-sibirici* used in this study. ^*b*^No data recorded in previous studies by Cummins (1971); Wang and Wei (1983), and Wang and Zhuang (1998), who only reported that the pedicle of teliospores was very short, described only by texts. ^*c*^No data available in the study by the three books above, which described that the length of the teliospore pedicel was the similar to that of teliospores.*

**FIGURE 8:**
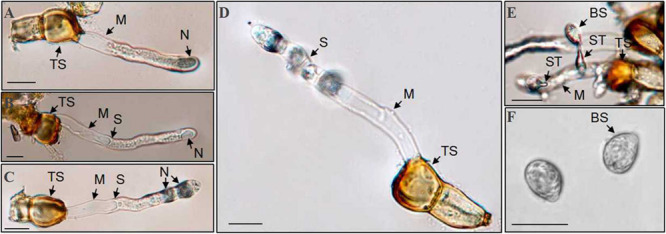
*Puccinia achnatheri-sibirici* teliospores (TS) germinated to produce basidiospores on the water agar medium. **(A)** Migration of nucleus (N) and cellular contents from the top cell of a two-celled teliospore (TS) into the top of a metabasidium (M) at the early stage of teliospore germination. **(B)** Formation of a septum (S) separated the top of a metabasidium (M) containing the nucleus (N) from the vacant basal cell of the metabasidium developed from a two-celled teliospore (TS). **(C)** Meiosis I showing formation of two daughter nuclei (N) at the top of a metabasidium (M) derived from a teliospore (TS). **(D)** Meiosis II showing formation of a four-celled metabasidium (M) separated by four speta (S). **(E)** A basidiospore (BS) growing at the tip of a sterigmata (ST) developed from a metabasidium (M). **(F)** Morphological single-celled and ovoid-shaped basidiospores (BS). Bars = 50 μm.

**FIGURE 9:**
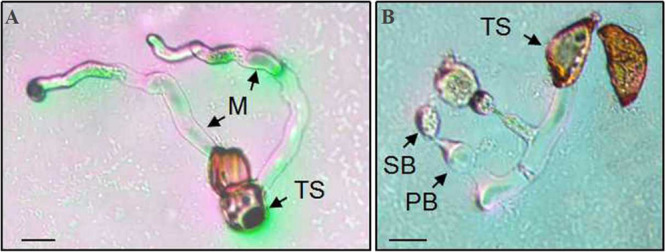
A *Puccinia achnatheri-sibirici* teliospore (TS) showing each of two cells could germinate to produce a metabasidium. Panel **(A)** showing that each cell of a two-celled teliospore (TS) of *Puccinia achnatheri-sibirici* can germinate to produce a metabasidium (M). Bars = 50 μm. **(B)** Formation of secondary basidiospores (SB) at the tips of primary basidiospore (PB). Bars = 50 μm.

Teliospores started to germinate at 4 h after being transferred on the surface of the water agar, producing a tube-shaped metabasidium (promycelium) with migration of cellular contents and the nucleus from a cell of the *P. achnatheri*-*sibirici* two-celled teliospore into the top of the metabasidium (Figure 8A). With the extension of the metabasidium, a septum formed inside the metabasidium, separating from cellular contents and the nucleus from the basal cell (Figure 8B). Two daughter nuclei formed inside the top of the metabasidium after meiosis I and meiosis II continued to form a four-celled metabasidium separated by four speta inside (Figures 8C,D). A sterigma produced from each of four cells of a metabasidium and a basidiospore formed at the tip of the sterigma (Figure 8E). Basidiospores were tiny, ovoid in shape, and single-celled with the size of 13.4 (11.4–15.9) μm in length and 9.5 (7.4–11.5) μm in width based on measurement data of 102 basidiospores (Figure 8F). A metabasidium can be produced from each of two cells of a teliospore of *P. achnatheri-sibirici* (Figure 9A). Occasionally, formation of a secondary basidiospore at the tip of a primary basidiospore can be observed (Figure 9B).

## Discussion

We demonstrated that *P. achnatheri-sibirici* is a heteroecious, macrocyclic rust fungus having five spore stages and *B. shensiana* can serve as an aecial host. This species, infecting gramineous grass *A. extremiorientale*, differs from any other rust species on *Achnatherum* spp. recorded (Cummins, 1971; Wang and Wei, 1983; Wang and Zhuang, 1998).

We also have tested other four *Berberis* species, including *Berberis phanera*, *Berberis aggregata*, *Berberis soulieana*, and *Berberis cavaleriei*, by inoculation using *P. achnatheri-sibirici* basidiospores developed from teliospores in the same conditions used in the present study. Based on three repeat tests, only two of them, *B. cavalerie* and *B. aggregate*, can be infected by *P. achnatheri-sibirici* and the rust fungus completed the whole sexual cycle on the two *Berberis* species. However, *B. phanera* and *B. soulieana* were not infected by *P. achnatheri-sibirici*, showing resistance to the rust fungus. In China, there are rich *Berberis* species resources and more than 250 *Berberis* species were recorded in the Chinese botanic monograph Flora of China (English E-version at website http://www.iplant.cn/foc/). This indicated that more *Berberis* species could serve as potential aecial hosts for the rust fungus.

Various *Achnatherum* species are infected by multiple rust fungi in the genus *Puccinia*, including *P. achnatheri-sibirici*. Some of them are morphologically similar to *P. achnatheri-sibirici* and have known aecial hosts. For example, *P. achnatheri-inebriantis* Z. Y. Zhao, a similar rust species to *P. achnatheri-sibirici*, was distinguished from the latter by its teliospores that have a long-stalk cell and a thick cell wall on the top of the upper cell. However, the aecial host of *P. achnatheri-inebriantis* is *Aster altaicus* Willd., which is not closely related to *Berberis* (Zhao, 1985).

Sexual reproduction is a vital stage of the life cycle of macrocyclic, heteroecious species of rust fungi and plays roles in surviving unfavorable climatic conditions, providing initial inoculum and/or generating diverse populations (Zhao, 2017). Barberry serves as an aecial host for several *Puccinia* species. Different formae speciales of *P. graminis* complete the sexual hybridization on barberry (de Bary, 1866; Johnson et al., 2006). Thus, barberry serves as a bio-platform for virulence recombination by nuclear heteromixis and reassembly at the sexual stage, which can greatly increase the virulence diversity of the progeny population after sexual hybridization. It is not clear whether *P. achnatheri-sibirici* could carry out sexual hybridization with other *Puccinia* species on susceptible *Berberis* species as their common aecial hosts. Recently, under laboratory conditions, we tried to make inter-species cross between *P. achnatheri-sibirici* and *P. striiformis* f. sp. *tritici* on barberry which successfully produced aeciospores. Currently, we are conducting studies to characterize the aecial and uredinial progenies.

In China, there are 1150 species belonging to 193 genera in Gramineae, which could be infected by 129 rust species (Wang and Wei, 1983). Sexual stages have been reported for many species in the genus *Puccinia*. However, sexual stages have not been found for most of the species (Mains, 1933; Wang and Wei, 1983; Wang and Zhuang, 1998). Therefore, we need to persevere in collecting *Puccinia* species infecting the grasses, belonging to hemicyclic rust fungi, to identify existence or absence of these rust parasites. This will be beneficial in understanding the diversity of the rust fungi in *Puccinia* and providing an insight into the evolution of *Puccinia* species.

Germination of basidiospores can be conducted in two ways. The basidiospore produces a germ tube directly or undergoes a process like sterigma, in which the sporidium (secondary basidiospore) is borne on the tip, called indirect germination (repetitive germination). It depends on environment conditions and the surface of hosts (Gold and Mendgen, 1991). Repetitive germination is common as the basidiospores survive in water or on the water agar (Mims and Richardson, 1990). Some scientists consider that it is an escape mechanism facing unseemly conditions (Alexopoulos et al., 1996).

Formation of secondary basidiospores in some of rust fungi is completed in different environmental conditions and temperatures. Biological phenomena on the production of secondary basidiospores derived from germinated teliospores have been reported in water in *Gymnosporangium fuscum* (Metzler, 1982), or in both water and air in *Gymnosporangium*, *Coleosporium*, *Cronartium*, *Phragmidium*, *Puccinia*, *Melampsora*, and *Uromyces appendiculatus* (Bauer, 1987; Freytag et al., 1988). This also was observed on the water agar in *Melampsora larici-populina* (Gong et al., 2015), *Coleosporium phellodendri* (Wei et al., 2013), and *P. achnatheri-sibirici* observed in this study. Secondary basidiospores can be produced at a range of temperatures and are reported in *Melampsora larici-populina* (Gong et al., 2015), *C. phellodendri* (Wei et al., 2013), and *M. larici-tremulae* (Shang and Pei, 1984). In addition, formation of tertiary spores has been reported in some rust fungi (Shang and Pei, 1984; Wei et al., 2013; Gong et al., 2015), but not in *P. achnatheri-sibirici*.

The pathogenicity of secondary basidiospores similar to or stronger than those of primary basidiospores has been reported in *Cronartium quercuum* f. sp. *fusiforme* (Roncadori, 1968) and *Gymnosporangium haraeanum* (Yu, 2009).

This study demonstrates that *Berberis* spp. are able to serve alternate hosts of *P. achnatheri-sibirici* on *A. extremiorientale* and showed that this rust species are heteroecious, macrocyclic rust fungi with five types of spores. The study also expands the knowledge on rust fungi that have *Berberis* species serving as alternate hosts.

## Data Availability Statement

The datasets generated for this study can be found in the National Center for Biotechnology Information. MN913585, MN913586, MN913587, MN915132, and MN915138.

## Author Contributions

JZ found the material of this study in the field and designed the study. ZK provided project support and platform for this study. XM, YL, XT, ZD, and QL performed the experiments. XM performed the statistical analysis. XM and JZ wrote the first draft of the manuscript. All authors contributed to the article and approved the submitted version.

## Conflict of Interest

The authors declare that the research was conducted in the absence of any commercial or financial relationships that could be construed as a potential conflict of interest.

## Footnotes

1 http://www.ncbi.nlm.nih.gov/

2 http://www.mybiosoftware.com/bioedit-7-0-9-biological-sequence-alignment-editor.html

3 https://blast.ncbi.nlm.nih.gov/Blast.cgi
