# Supplementary material for: Identification of Berberis spp. as Alternate Hosts for Puccinia achnatheri-sibirici Under Controlled Conditions and Morphologic Observations of Sexual Stage Development of the Rust Fungus
Source: Front Microbiol. 2020 Jun 25;11:1278. doi: 10.3389/fmicb.2020.01278 (PMC7330019; doi:10.3389/fmicb.2020.01278)
Supplement: Supplementary file 1 [file Data_Sheet_1.docx]

Supplementary Material

# Supplementary Data

The datasets analyzed for this study can be found in the NCBI GeneBankwww.ncbi.nlm.nih.gov/.

>Teliospores of *Puccinia achnatheri-sibirici* (accession Number: MN913585)

CGACGGCAGTGTGAACCTGCAGAAGGATCATTATTAAAAGAACTAGAGTGCACTTTATTGTGGCTCGACCCTTTTAACAACTCACCCAAACACTTTTAAGACTTGGTTGCATGATTTGAAAAAGTCATTGCAATTGAGTAGACGTAACTTCTTTATTGAATGTTGCATTACCCCTTTTTATTTTTTAATTTTTTTTAAACACAAGTTGAAATGAATGTAACCAAACCTTTAATTATAAATAACTTTTAACAATGGATCTCTAGGCTCTCACATCGATGAAGAACACAGTGAAATGTGATAAGTAATGTGAATTGCAGAATTCAGTGAATCATCGAATCTTTGAACGCACCTTGCGCCTTTTGGTATTCCAAAAGGCACACCTGTTTGAGTGTCATGAAACCCTCTCATTAAATAATTTTAATTAATTATTTTCAATGGATGTTGAGTGTTGCTGTAATTAGCTCACTTTAAATATATAAGTCACTTTTCAATAAGTTGGATTGACTTGGTGTGTAATAATTTTATCATCACATTAAGGAAAGTAGTAATACTGCCATCTTGTTTTTGAAAGGAGACTCCT

>Aeciospores of *Puccinia achnatheri-sibirici* (accession Number: MN913598)

TCATTATTAAAAGAACTAGAGTGCACTTTATTGTGGCTCGACCCTTTTAACAACTCACCCAAACACTTTTAAGACTTGGTTGCATGATTTGAAAAAGTCATTGCAATTGAGTAGACGTAACTTCTTTATTGAATGTTGCATTACCCCTTTTTATTTTTTTATTTTTTTTTTTAAACACAAGTTGAAATGAATGTAACCAAACCTTTAATTATAAATAACTTTTAACAATGGATCTCTAGGCTCTCACATCGATGAAGAACACAGTGAAATGTGATAAGTAATGTGAATTGCAGAATTCAGTGAATCATCGAATCTTTGAACGCACCTTGCGCCTTTTGGTATTCCAAAAGGCACACCTGTTTGAGTGTCATGAAACCCTCTCATTAAATAATTTTGATTAATTATTTTCAATGGATGTTGAGTGTTGCTGTAATTAGCTCACTTTAAATATATAAGTCACTTTTCAATAAGTTGGATTGACTTGGTGTGTAATAATTTTATCATCACATTAAGGAAAGTAGTAATACTGCCATCTTGTTTTTGAAAGGAGACTCCTAAAAAACCCCCTCTTTTTATTTTTTAAAACCTCAAATCAGGTGGGACTACCCGCTGAACTTAAGCAT

>Uredinioospores of *Puccinia achnatheri-sibirici* (accession Number: MN913586)

TATGAGTGACTTTATTGTGGCTCGACCCTTTTAACAACTCACCCAAACACTTTTAAGACTTGGTTGCATGATTTGAAAAAGTCATTGCAATTGAGTAGACGTAACTTCTTTATTGAATGTTGCATTACCCCCCCTCCTTTTTTATTTTATTTTTTTAAACACAAGTTGAGATGAATGTAACCAAACCTTTAATTATAAATAACTTTTAACGATGGATCTCTAGGCTCTCACATCGATGAAGAACACAGTGAAATGTGATAAGTGATGTGAATTGCAGAATTCGAAGAATCATCGAATCTTTGAACGCACCTTGCGCCTTTTGGTATTCCAAAAGGCACACCTGTATGAGTGTCATGAAACCCTCTCATTAAATAATTTTGATTAATTATTTTCGATGGATGATGAGTGTTGCTGTAATTACCTCACTTTAAATATATAAGTCACTTTTCAATAAGTAGGATTGACTTGGGGTGTAATAATTTTATCATCACATTAAGGAAAGTAGTAATACTGCCATCTTGTTTTTGAAAGGATACTCCTAAAC

>*P. brapychodii* H-KI (accession Number:MN915132)

AATGCGCGGATCTTCAGAGATTTGAACCTGCAGAAGGATCATTATTAAAAGAACTAGAGTGCACTTTATTGTGGCTCGACCCTTTTAAACAACTCACCCAAACACCATTAAGACTTGGTTGCATGATTTGAAAAAGTCATTGCAATTGAGTAGACGTAACTTCTTGATTGAATGTTGCATTACCCCCCCCTTTAATTTTTTTTATTTTTATAAATATAAACACAAGTTTAAATGAATGTAACCAAACCTTTAATTATAAATAACTTTTAACAATGGATCTCTAGGCTCTCACATCGATGAAGAACACAGTGAAATGTGATAAGTAATGTGAATTGCAGAATTCAGTGAATCATCGAATCTTTGAACGCACCTTGCGCCTTTTGGTATTCCAAAAGGCACACCTGTTTGAGTGTCATGAAACCCTCTCATTAAATAATTTTGATTAATTATTTTCAATGGATGTTGAGTGTTGCTGTAATTAGCTCACTTTAAATATATAAGTCACTTTTCAATAAGTTGGATTGACTTGGTGTAATAATTTTATCATCACATCAAGGAAAGTAGTAATACTGCCATCTTGTTTCTGAAAGGAGACTCCTAAAAAACCCTCTTTTTTTTTTAAGACCTCAAATCAGGTGGGACTACCCGCTGAACTTAAGCATATCAATAAGC

>*P. brapychodii* F CO (accession Number:MN913138)

TAGACGGCAGTGATTAGTGAACCTGCAGAAGGATCATTATTAAAAGAACTAGAGTGCACTTTATTGTGGCTCGACCCTTTTAAACAACTCACCCAAACACCATTAAGACTTGGTTGCATGATTTGAAAAAGTCATTGCAATTGAGTAGACGTAACTTCTTGATTGAATGTTGCATTACCCCCCCCTTTAATTTTTTTTTATTTTTATAAATATAAACACAAGTTTAAATGAATGTAACCAAACCTTTAATTATAAATAACTTTTAACAATGGATCTCTAGGCTCTCACATCGATGAAGAACACAGTGAAATGTGATAAGTAATGTGAATTGCAGAATTCAGTGAATCATCGAATCTTTGAACGCACCTTGCGCCTTTTGGTATTCCAAAAGGCACACCTGTTTGAGTGTCATGAAACCCTCTCATTAAATAATTTTGATTAATTATTTTCAATGGATGTTGAGTGTTGCTGTAATTAGCTCACTTTAAATATATAAGTCACTTTTCAATAAGTTGGATTGACTTGGTGTAATAATTTTATCATCACATCAAGGAAAGTAGTAATACTGCCATCTTGTTTCTGAAAGGAGACTCCTAAAAAACCCTCTTTTTTTTTTAAGACCTCAAATCAGGTGGGACTACCCGCTGAACTTAAGCATATCAATAAGC
